# ADAM-Plant: A Software for Stochastic Simulations of Plant Breeding From Molecular to Phenotypic Level and From Simple Selection to Complex Speed Breeding Programs

**DOI:** 10.3389/fpls.2018.01926

**Published:** 2019-01-09

**Authors:** Huiming Liu, Biructawit Bekele Tessema, Just Jensen, Fabio Cericola, Jeppe Reitan Andersen, Anders Christian Sørensen

**Affiliations:** ^1^Center for Quantitative Genetics and Genomics, Department of Molecular Biology and Genetics, Aarhus University, Aarhus, Denmark; ^2^Rijk Zwaan, De Lier, Netherlands; ^3^Nordic Seed, Odder, Denmark

**Keywords:** stochastic simulation, genomic selection, plant breeding, breeding program, wheat

## Abstract

Making decisions on plant breeding programs require plant breeders to be able to test different breeding strategies by taking into account all the crucial factors affecting crop genetic improvement. Due to the complexity of the decisions, computer simulation serves as an important tool for researchers and plant breeders. This paper describes ADAM-plant, which is a computer software that models breeding schemes for self-pollinated and cross-pollinated crop plants using stochastic simulation. The program simulates a population of plants and traces the genetic changes in the population under different breeding scenarios. It takes into account different population structures, genomic models, selection (strategies and units) and crossing strategies. It also covers important features e.g., allowing users to perform genomic selection (GS) and speed breeding, simulate genotype-by-environment interactions using multiple trait approach, simulate parallel breeding cycles and consider plot sizes. In addition, the software can be used to simulate datasets produced from very complex breeding program in order to test new statistical methodology to analyze such data. As an example, three wheat-breeding strategies were simulated in the current study: (1) phenotypic selection, (2) GS, and (3) speed breeding with genomic information. The results indicate that the genetic gain can be doubled by GS compared to phenotypic selection and genetic gain can be further increased considerably by speed breeding. In conclusion, ADAM-plant is an important tool for comparing strategies for plant breeding and for estimating the effects of allocation of different resources to the breeding program. In the current study, it was used to compare different methodologies for utilizing genomic information in cereal breeding programs for selection of best-fit breeding strategy as per available resources.

## Introduction

The goal of most plant breeding programs is to hybridize and select best elite lines or varieties with the best combination of desired characteristics, viz., yield-attributing traits, quality traits, and insect and pest resistance (Collard and Mackill, 2008). The success of selection based on yield-related traits has been attributed to classical PS methods by which superior individuals are selected based on their individual phenotypic performance or combined index. Besides, with decreasing costs of SNP genotyping, MAS was widely used in particular for biotic and abiotic stress resistant traits (Jonas and de Koning, 2013; Ragimekula et al., 2013). However, the success of selection can either be limited with PS if the trait under selection has low heritability or with MAS if the trait is govern by many quantitative loci (QTLs) with small effects. The introduction of GS has provided the opportunity to overcome these limitations. GS refers to selection decisions based on GEBVs, which are calculated as the sum of effects of dense genetic markers in LD with one or more QTLs across the entire genome (Meuwissen et al., 2001; Meuwissen, 2007; Heffner et al., 2009). The key advantages of integrating of GS in plant breeding decisions are an increase in genetic gain per breeding cycle and a reduction in the length of the breeding cycle (Crossa et al., 2017). In addition to applying GS, there are many other factors, which also affect the genetic gain in a plant-breeding program. These factors include breeding objectives, experimental design (e.g., plot size and number of replicates per family), selection strategy (e.g., individual/family selection and recurrent selection) and biological aspects (e.g., mode of pollination, self-incompatibility, heritability and genotype–environment interactions). Consideration of all these factors means that the number of alternative breeding programs can be numerous. The choice of breeding program is based on how plant breeders are able to test the consequences of selected alternatives. Comparing alternative breeding strategies in large-scale field experiments can be labor-intensive in terms of time and effort needed. Instead of field experiments, simulation studies can serve as an efficient means to model different breeding strategies and predict their performance. Moreover, simulation studies can provide us with a deep understanding on the impact of different factors on the genetic gain and other variables of interest (e.g., selection accuracy and genetic variance) of breeding programs, which facilitates the development and choice of better breeding programs. From a methodological point of view, quantifying the expected genetic gain of breeding programs can be done using either stochastic simulation or deterministic methods. In the cases where a number of factors need to be accounted for, however, it may be difficult to derive accurate deterministic methods. The advantage of stochastic simulation in this situation is that, it can be used to simulate an entire population of individual plants, so that one can mimic the actual artificial plant breeding programs in any detail desired. This enables stochastic simulation to be able to provide very precise prediction of consequence of alternatives. Hence, a tool that is capable of simulating a large range of practical breeding programs with sufficient feasibility and flexibility needs to be developed.

Many software packages are available to design alternative plant breeding programs. Sun et al. (2011) provided a summary of available software packages with focus on the diversity in the features, functionality and underlying assumptions in each program. For instance, GREGOR predicts the average outcome of mating or selection under specific assumptions about gene action, linkage, or allele frequency (Tinker and Mather, 1993); Qu-Gene models^1^, such as QuLine, QuHybrid, and QuMARs are capable to simulate simple to complex genetic models mimicking line breeding programs, including conventional selection, MAS and GS (Wang et al., 2003; Wang and Dieters, 2008a,b; Li and Wang, 2011; Wang, 2011). Faux et al. (2016) has developed a software, which integrates biotechnologies such as DHs and gene editing and allow simulating sequence data. It can be used to simulate a wide range of possible scenarios using GS in the breeding programs. While these simulation tools are useful in improving the efficiency of different selections strategies, details regarding breeding cycles in actual breeding programs applied in the field were not considered in most of these simulation software packages. They only allow simulating one breeding program running at each time step, ignoring the fact that in practice every year a new breeding scheme starts with a similar experimental set-up as the previous breeding cycle, which also enables interactions between breeding cycles.

The current work introduces ADAM-plant, which is a software for simulating breeding programs with overlapping breeding cycles for self-pollinated (e.g., line breeding of inbred varieties) and cross-pollinated crop plants (e.g., breeding of synthetic varieties)and for application of new technologies such as speed breeding. It was developed from an existing computer program named ADAM, which can simulate a large range of breeding programs for animals using stochastic simulation (Pedersen et al., 2009). ADAM-plant models the full complexity of actual breeding programs and allows users to generate sequence data, perform GS using different genomic prediction models and simulate genetic × environment interactions using multiple trait approach for plant breeding. These features enable ADAM-plant to be a tool for both research purpose and plant breeding companies to test and develop breeding strategies with consideration of various aspects:

(1) It allows users to simulate overlapping breeding cycles with a new cycle starting at each time step. In this paper, each time step represents a reproductive step with different actions such as selection, testing or replication being carried out at specific time steps. The simulation of overlapping breeding cycles allows early generations in one cycle to be used as parents in a new cycle before the parental cycle is finished.
(2) It allows users to simulate different breeding strategy (e.g., recurrent selection and speed breeding) for different stage of the breeding program. For instance, there are many options for different actions in each time step i.e., selected plants can be genotyped, phenotyped, reproduced and/or have germplasm stored.
(3) It allows different units of selection. The selection unit can be population, within family, or entire family. The family can be full-sib family, half-sib family or defined by a group of parents (e.g., a set of parents or a set of plants used in a poly-cross to create synthetics) in an earlier generation.
(4) It allows accounting for plot size (number of plants grown), that can be used for mimicking different planting density in different generations.

This paper describes the simulation method and working process of ADAM-plant in different plant breeding applications with an emphasis on its main characteristics, component elements and computational performance using a couple of examples of wheat-breeding programs.

## Materials and Methods

**FIGURE 1:**
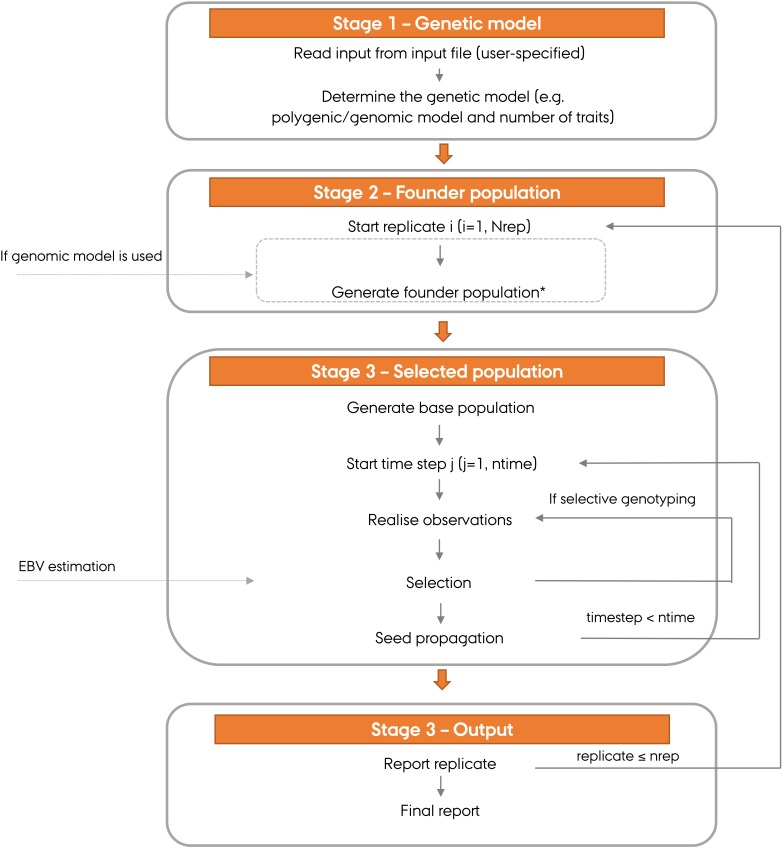
An of ADAM-plant working pipeline. ^∗^The replicates of selected populations can start either from the same founder population or from a unique founder population in each replicate. Alternatively, the base population can consist of phased genotypes from real or simulated individuals.

An overview of ADAM-plant working pipeline is presented in Figure 1. It consists of four stages:

(1) The genetic model, which is the method used to generate breeding values, is specified by the user.
(2) A founder population is simulated if genomic model with LD between QTLs and markers is used. This creates desired LD in the genome. This founder population is used as the basis for subsequent stages.
(3) The selected population is generated. It initiates with a base population that is assumed unrelated based on the pedigree and followed by selection in subsequent generations. The user needs to specify the number of replicates of the selected population, selection strategy used for each selection stage and the type of propagation used for seed reproduction. If genomic model is used, the simulation of the selected population can initiate from the same or a unique founder population, depending on the choice made by the user.
(4) The output variables are recorded and analyzed. Not all output variables are required in any simulation. The user specifies what to output (e.g., genetic gain, genetic variance and accuracy of EBVs) in each time step. These results are also summarized when more than one replicate have been obtained. If desired all the data generated on the molecular as well as phenotypic level in each time step can also be saved for further analysis.

### Genetic Model

There are mainly two models available to generate breeding values for single and multiple traits.

(1) Infinitesimal model, mimicking a polygenic makeup.
(2) Genomic model with LD between QTLs and markers where individual QTL and markers are simulated.

When a genomic model is used, it is necessary to simulate founder population or read in individual-level genome-wide SNP/sequence data either collected from breeding programs under investigation or simulated. This step is skipped when an infinitesimal model is used.

### Founder Population

When a genomic model is used, a founder population is generated by default using a Fisher-Wright inheritance model in order to establish recombination-drift-mutation-selection equilibrium and create initial LD among markers and QTLs (Fisher, 1930; Wright, 1931). The maternal and paternal haploid genomes of founder individuals are simulated by considering discrete generations, random mating and three evolutionary forces: mutation, drift and selection based on the fitness of the individuals. The user needs to define the number of paternal (*N*_pat_) and maternal (*N*_mat_) founder plants, the number and length of chromosomes (*N*_chrom_), the number of loci (*N*_loci_) on each chromosome and the number of founder generations (*NG_founder_*). Expansion and contraction of the founder population are allowed in order to generate different LD structures e.g., long/short-range LD (Schopp et al., 2017). The plants are randomly mated without selfing for *NG*_founder_ discrete generation producing (*N*_pat_ + *N*_mat_) offspring. In the first generation of founder population, a total of *N*_loci_ are evenly distributed across *N*_chrom_ genomes. The user specifies *N* so that every *Nth* locus harbors a QTL that coded for the trait under selection. The remaining loci are genetic markers. The user needs to specify the mutation rate, which is the probability of mutation occurring at each locus of genotype. Bi-allelic polymorphism at each locus is generated with this specified mutation rate. An additive effect for the mutant allele at each QTL is sampled from an exponential distribution. The additive effects are assumed to be negative probability 0.9 by default because the exponential distribution only generates positive values. The additive effects of the wild-type alleles are zero. Selection is introduced by culling and resampling 5% of plants with lowest TBV by default. The TBV of the ith plant in the founder population, *g*_i_ , is calculated as $g_{i}=\Sigma_{j=1}^{n_{QTL}}$, where *n*_QTL_ is the number of QTLs across the genome, x_ij_ is the number of copies of the mutant allele that plant *i* inherited at *jth* QTL (*x_ij_* = 0, 1, 2), and *g_i_* is the additive effect of mutant allele at *jth* QTL. Each offspring inherits marker and QTL alleles from their parents following Mendel’s rules allowing for recombination. ADAM-plant samples the number of crossovers from a Poisson distribution with mean number of crossovers equal to the length of the chromosomes in Morgan and then the crossovers are placed randomly along the chromosome. Alternatively, the user can also generate and store their genome of the founder population externally or use real genome and import them into ADAM-plant. The founder plants in generation *NG_founder_* or the stored genome are pooled for the subsequent sampling of base population.

#### Base Population and Trait

When an infinitesimal model is used, TBV of individuals in the base population are sampled from a normal distribution with user-specified additive genetic variance. When genomic model is used, the genotype of each base plant is sampled from the pool of chromosomes in generation *NG_founder_* of the founder population. For chromosome *k* (*k* = 1…*N*_chrom_), two chromosomes are randomly sampled without replacement from the *k*th pool of chromosomes. The sampled chromosomes are replaced before the next base plant is sampled. When the plants in a base population are assumed inbred lines (self-pollinated), then the second haplotype of a base plant is set to be identical to the first haplotype. The user needs to define the genetic variance $\sigma_{g}^{2}$ of the trait. The additive effects of the mutant alleles at the segregating QTLs are standardized so that the total additive genetic variance for the trait under selection is $\sigma_{g}^{2}$. The environmental effect for each individual plant is sampled from a normal distribution N(0,$\sigma_{e}^{2}$), which ensures that $h^{2}=\frac{\sigma_{g}^{2}}{\sigma_{g}^{2}+\sigma_{e}^{2}}$. As a result, the phenotypes of each individual is a sum of genetic and environmental effects. In some situations, instead of _*h*^2^_ , the user may have prior knowledge about heritability of plot phenotypes (plot heritability; $h_{plot}^{2}$) in a certain generation (Nyquist, 1991; Ogunniyan and Olakojo, 2014), which is equal to the square of the correlation between the sum of TBV of individuals and sum of the phenotypes of individuals in the plots. Under the assumption of Hardy-Weinberg Equilibrium $\sigma_{e}^{2}$ can be estimated using the equation:

$$\sigma_{e}^{2}=\frac{2N_{s}H\sigma_{g}^{2}\left(1-h_{plot}^{2}\right)}{h_{plot}^{2}}-\left(1-H\right)\sigma_{g}^{2}$$

where *H* is the expected frequency of homozygosity in the generation and *N*_s_ is the number of plants grown per plot. Then in the situation where selection is performed, the user needs to calibrate $\sigma_{e}^{2}$ according to this equation by trial and error so that the realized $h_{plot}^{2}$ is equal to the desired $h_{plot}^{2}$.

When genomic model is used, ADAM-plant also allows users to trace the contribution of each plant in the base population to following generations and infer IBD status relative to the base plants. This is done by assigning equidistant IBD markers across the genome. These IBD markers are assigned unique alleles to each base plant, but not involved in selection. Within each locus of a descendant, each IBD marker allele could be traced directly back to the base plants from which it was derived (Liu et al., 2017). True inbreeding of each individual, which measures the level of inbreeding of the whole genome, is calculated using these IBD markers as the proportion of IBD markers that are identical by descent within an individual.

ADAM-plant allows simulation of multiple traits. Taking finite-locus model as an example, the correlated traits are characterized by the same set of QTLs, although some of these QTLs may have near-zero effect on one or more traits. The QTL effects on multiple traits are sampled from a standard multivariate normal distribution with a user-specified matrix of additive genetic variance and covariance between the traits.

### Simulation of Breeding Programs

#### Cycles

**FIGURE 2:**
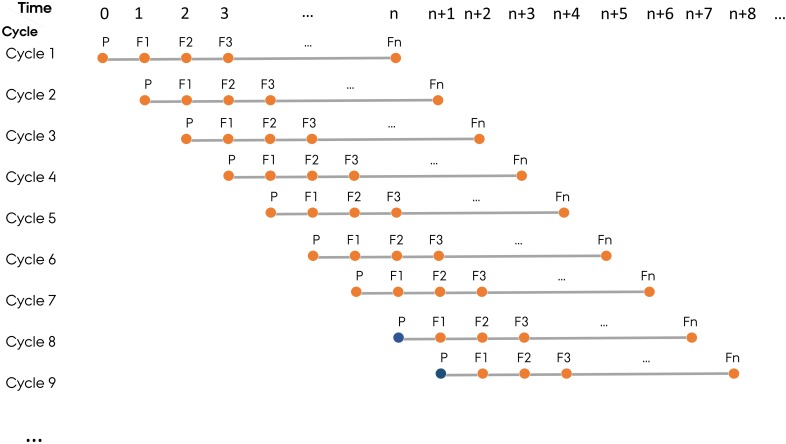
The structure of simulated plant breeding programs, which mimics the practical plant breeding programs with parallel breeding cycles starting at each time step. In each cycle, P represents parental lines and F1–Fn represent generation 1 to n. Fn is the last generation of a breeding cycle. The base population (filled orange circle) in the first n-1 cycles are established by randomly sampling the two haplotypes from the founder population. From cycle N_gen_ onward, however, the base population (filled blue circle) is sampled from germplasm of selected individuals in the user-defined generation(s) in the previous cycles.

To mimic the structure of commercial plant breeding programs, ADAM-plant allows simulation of parallel breeding cycles with a new cycle starting at each time step. In this paper, a generation represents a distinct phase where meiosis and fertilization have occurred in the context of a breeding cycle. The user needs to define the last generation of a breeding cycle, which is the generation where new lines will be released to relevant markets. Normally, a cycle in a cereal breeding program can last up to 8 generations (*n* = 8) from the first parental lines to the final selection of a new elite line, but other crops may have a different length of a breeding cycle. The base population in the first *n* - 1 cycles are established by randomly sampling two copies of the genome of all the base plants from the founder population. From cycle *n* onward, however, the base population is built using the germplasm stored from user-specified generation(s) in the previous cycles (Figure 2).

#### Speed Breeding

**FIGURE 3:**
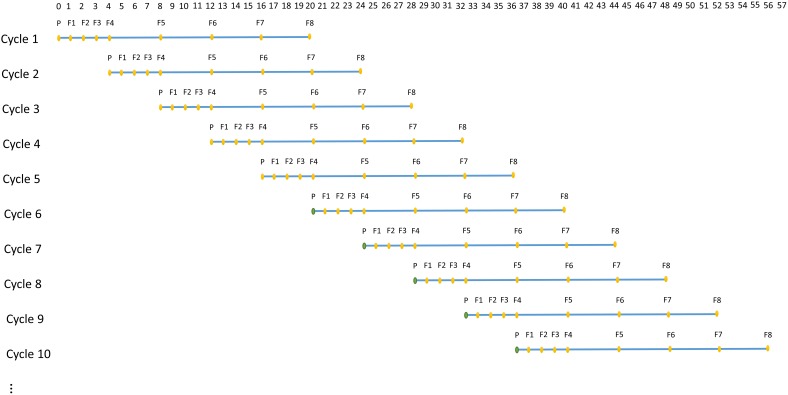
An example of structure of simulated speed breeding programs for crop plants, which mimics the practical wheat breeding programs with parallel breeding cycles starting at each time step. In each cycle, P represents parental lines and F_1_ to F_8_ represents generation 1 to 8. The reproduction of the first 4 generations (F1–F4) are assumed to achieve within a year, whereas the reproduction of remaining generations takes 1 year each. The base population (filled orange circle) in the first 5 cycles are established by randomly sampling the two haplotypes from the founder population. From cycle 6 onward, however, the base population (filled green circle) is sampled from germplasm of selected individuals the user-defined generation(s) in the previous cycles.

A technology that allows rapid generation advancement, called “Speed breeding,” can be used to achieve 4–6 generations of wheat per year (Alahmad et al., 2018; Watson et al., 2018). Speed breeding utilizes extended period of light to accelerate growth rate of a plant, which greatly shortens generation time and accelerates genetic improvement. ADAM-plant enables the simulation of speed breeding by allowing the user to specify the number of time steps or years each generation takes. For instance, in each cycle, the first 4 generations can be assumed to achieve within a year instead of 4 years (Figure 3).

#### Phenotyping

As mentioned above, the phenotype of the trait for the *ith* base plant, *y_i_*, was calculated as *y_i_* = *g_i_* + *e_i_*, where *g_i_* is the base plant’s true additive-genetic value and *e_i_* is its residual environmental value. In descendant generations, the alleles at QTL and marker positions were sampled according to principles of Mendelian inheritance, and the phenotypes of plants in these generations are also a sum of *g* and *e*.

The user needs to specify at which generations and which selection stages the phenotypic record of each trait is realized. The user also needs to specify the number of observations, which represents the number of replicates recorded for the trait(s) of interest and number of plots grown per family in a user-specific selection stage. For instance, if there are three observations for a trait, then there are three plots for each family.

#### Breeding Goal and Economic Value

The user needs to specify the economic values for each trait to calculate true aggregate-breeding value. The aggregate-breeding value is calculated by weighting TBV of each trait by its economic value. The user can also specify different economic values on each trait in each selection stage.

#### Selection

Selection is carried out in each time step. Selection is carried out on single or multiple traits by threshold selection or truncation selection. Threshold selection is based on the phenotypic observation(s) with associated threshold. Truncation selection is based on one of the following criteria: phenotype, BLUP (based on phenotype and pedigree information), GBLUP, single-step GBLUP when only parts of plants are genotyped or phenotyped (Legarra et al., 2009; Christensen and Lund, 2010) or Bayesian models (Yu and Meuwissen, 2011). The breeding values are estimated using DMU version 6 (Madsen and Jensen, 2013), which is a package for analyzing multivariate mixed models, including prediction of breeding values.

ADAM-plant also allows users to carry out optimum-contribution selection (OCS), which maximizes long-term genetic gain while constraining inbreeding by constraining the relationship among selected parents (Wray and Goddard, 1994; Meuwissen, 1997). Optimum contribution selection allocates matings to selection candidates at time *t* by maximizing the function *U_t_*:

a^Ut(c) =c′a^−ωL2(c+Pv)′A(c+Pv)

where *c* is a *n* vector (*n* is the total number of plants in the population pedigree) of genetic contributions to the new cohort and the number of matings allocated to each candidate is a linear function of these contributions, a^ is a vector of EBVs, ω is the penalty applied to the average relationship of the current generation, *L* is the generation interval, *v* is a *k* vector of expected relative contributions to future age-class, *P* is a *n* × *k* matrix of contributions to future age-class of plants in the current generation, *A* is a *n* × *n* matrix of additive–genetic relationships or genomic relationships (Henryon et al., 2015). ADAM-Plant makes use of “EVA” to perform optimum-contribution selection (Berg et al., 2006).

Selection can be carried out in single or multiple stages in the population, on lines, within families, across families or on entire families, depending on the user-specified selection unit.

#### Seed Propagation

Before considering the type of seed propagation, the user needs to specify reproductive and life cycle characteristics of the population. Such characteristics include the reproductive age of the plant, the last generation of the breeding cycle and the generation at which germplasm is stored for later use.

There are four options for seed propagation: cloning, crossing, selfing, and DHs. Different types of seed propagation can be used in different propagation stages. Crossing can be performed either within families, across families or in the population. When crossing is used, the user needs to specify the maximum number of crosses where a plant can be used for crossing. Double-haploids are created by allowing recombination of the two haplotypes of an individual, randomly sampling one of the recombined gametes and then doubling this gamete to create a new diploid individual.

The user needs to specify the number of seeds generated from seed propagation at each selection stage. This is related to the selection unit. If the selection unit is population or within family, the number of seeds represents the number of seeds produced by each individual plant. If the selection unit is entire family, the number of seeds represents the number of seeds produced by the user-specified family.

### Output

The output files are generated and written to a user-specified output directory. Different variables for each generation and each time step can be output depending on the interest of the user. The description of the major variables is as follows:

(1) The phased haplotypes and genotypes of the simulated individuals for each chromosome, the position and allele frequencies of the QTLs and the markers, and the pedigree can be stored for each generation and each time step. This yields the opportunity to create simulated datasets from full scale and complex breeding programs e.g., to test new evaluation methods or models and to test methods for estimation of population parameters etc., In addition, it enables the user to develop summary statistics currently not included in the standard set of output.
(2) Estimated breeding values of every single plant can be stored if EBV is predicted for certain generation and time step.
(3) The average TBV and phenotypes for each trait can be stored for each trait, for each generation and each time step.
(4) Realized variance within- and between- families (families are specified by the user) can be calculated for each trait, for each generation and each time step.
(5) Accuracy of selection can be recorded if selection is performed at certain generation and time step. If the selection unit is population or within family, the accuracy is either the correlation between TBV and EBV under selection on EBV or the correlation between TBV and phenotypes under PS. If selection is on entire families, the accuracy is calculated as the correlation between the sum of TBV of individuals in the family and the sum of EBV/phenotypes in the family.
(6) Mean inbreeding computed based on pedigree information and, optionally, inbreeding based on IBD markers.

A log file is written and updated as the program is running. The log files show the detailed information of simulation process (e.g., the selection and mating strategy, number of selection candidates and the number of seeds produced) at each time step.

The user decides what genetic variables are saved. Not all outputs are required in any simulation. When all the replicates are complete, the mean and the standard deviation of all the genetic variables are also written. Plots showing development in means and standard deviations are written to pdf files as well.

### Availability

ADAM-Plant is written in Fortran 95. The program makes use of other programs for specific procedures: “Randlib90” to generate random numbers (Randlib.Version 90, 2002) and DMU to estimate breeding values (Madsen and Jensen, 2013); “EVA” for optimum contribution selection (Berg et al., 2006); and “IBD” to calculate identity-by-descent matrices (Thomsen, 2006). It has been developed under Linux on 32 and 64 bit based workstations. ADAM-plant is distributed as executable files and is free of charge for research purposes. Further information is available from http://adam.agrsci.dk.

### Examples

In the next section, the current study showed three examples of commercial wheat-breeding programs using a finite-locus model. The founder population for these programs is established based on stored real dataset containing 988 individual genomes collected from a commercial plant breeding company (Cericola et al., 2017). Data are from three breeding cycles, each consisting of 330 new F6 lines. Approximately 60 parental lines were crossed in the beginning of each breeding cycle, followed by five generations of selfing to produce the F6 lines. The number of markers were 9582 on the entire genome. As no QTL information was available in the data, among all the 9582 markers, 1039 loci that were randomly chosen across the genome were assumed as QTLs, and the remaining 8543 loci were assumed as markers for breeding value prediction. The real dataset help to make reasonable commercial breeding plans by considering the population structure and LD structure.

## Results

### Breeding Plan A. Breeding Program With Phenotypic Selection

**FIGURE 4:**
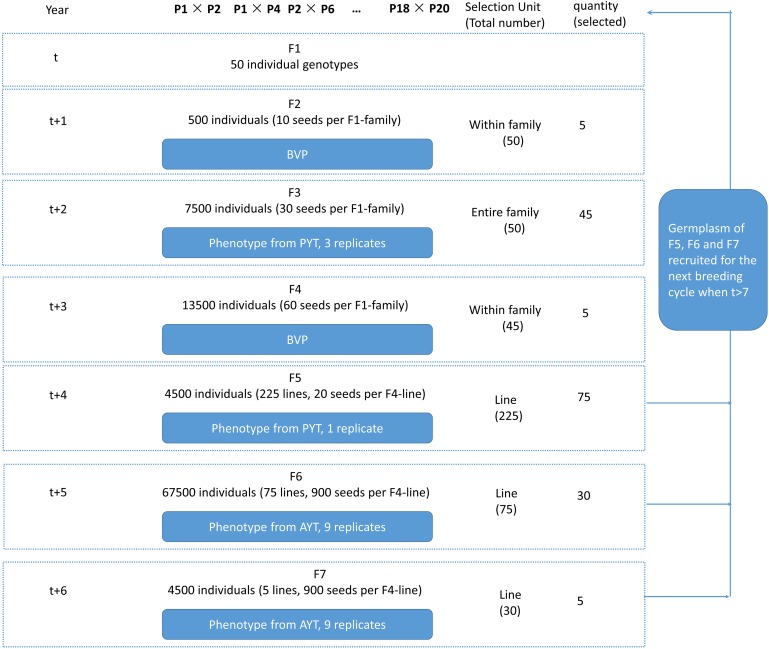
Overview of one cycle in the conventional breeding program with phenotypic selection. The F5, F6, and F7 were regarded as lines as the F4 individuals were used to create lines of single seed descent. Within each F4-line the individuals were genetically similar because of several rounds of selfing. For instance, there were 225 F4 lines as selection candidates in F5. Out of 225 lines, 75 lines were selected to produce F6. BVP, breeder’s visual preference yield; PYT, preliminary yield trial; AYT, advanced yield trials.

For breeding plan A, the current study simulated a 25-year commercial wheat-breeding program using phenotypic selection (Figure 4). The generation time is 1 year and a new breeding cycle started every year, so in total there were 25 breeding cycles initiated. Each breeding cycle was initiated by selecting 20 parental lines and completed at generation 8 (F8) after seven generations of selfing. In the first seven cycles, the genome of the 20 parental lines were randomly sampled from the 988 stored genomes without replacement and these 20 parental lines were used for crossing. Genotype-by-environment interaction was considered for the yield trait by taking F5 yield and F6/F7 yield as different traits. Therefore, three traits were simulated: BVP, YP in F5 and YAs in F6 and F7 (YA). The term “preliminary yield trial” means that the population is tested in one replicate in sparse field plots with reduced phenotyping. An important use of the YP, therefore is replication of seeds. The term “advanced yield trials” means that the population is tested when the amount of seeds for the population is sufficient to conduct multiple location trials in dense yield plots with possibilities for extensive phenotyping. The genetic variance of BVP, YP, and YA was set to 1 (standardized unit). Heritability of BVP was 0.1, and the plot heritability for YP and YA were 0.2 and 0.3 given prior knowledge of analysis using real dataset (Cericola et al., 2017). The current study investigated the consequence of the breeding program considering different correlations between the traits. Negative correlations between the traits were not considered. Four levels of correlation between YP and YA (0.1, 0.3, 0.5, and 0.7) and two levels of correlation between BVP and the other traits (0 or 0.1) were tested, resulting in eight scenarios. The selection was on different traits in different selection stages as follows:

(1) Parental lines P0: For the first seven cycles, 20 parental lines (P0) were randomly sampled from the founder population. For the remaining 18 cycles, the 20 P0 were sampled from the genotypes of selected F5, F6, and F7 in the previous cycles, assuming the germplasm of all the selected individual plants at these three generations were available. The 20 parental lines were allowed to be randomly crossed with each other, and the maximum times of crosses that one individual could be used were four. In total, a smaller subset of 50 out of possible 190 crosses were kept to produce F1.
(2) F1: In total, 50 F1 were generated. In reality, a single cross can actually produce a number of F1 individuals that are genetically identical. Therefore, for simplicity, only a single F1 individual was simulated for each cross. These 50 F1 were self-pollinated to produce 10 seeds (F2) each. From F2 to F4, the families were derived by selfing from their common ancestors at F1 generation and were denoted as F1-families.
(3) F2: Within family selection was conducted on F2. Within each of the 50 F1-families, out of 10 F2, the five highest-ranking individuals were selected based on BVP. The self-pollination of each selected F2 produced 30 seeds, resulting in 7500 F3 seeds in total.
(4) F3: Entire family selection was conducted based on the YP performance of F3 individuals in each F1-families. Each F1-family was assumed planted in three plots, and therefore, three replicates were simulated. In total, out of 50 families, 45 families with highest-ranking YP were selected and were self-pollinated to produce 60 seeds (F4) per F1 family (20 F4 per plot).
(5) F4: Within family selection was conducted for F4. Within each of the 45 F1-families, five highest-ranking individuals were selected based on BVP. So 225 F4 were selected in total. From now on, these F4 were used to create lines of single seed descent. Each F4 was self-pollinated to generate 20 seeds, resulting in 4500 F5. From F5 to F8, the selection were all based on the F4-lines.
(6) F5: Line selection was conducted based on the YP performance of all F5 individuals in each F4-line. Only one replicate was simulated for each F4-line. In total, 75 out of 225 lines with highest YP were selected and were self-pollinated to produce 900 F6 per line (100 F6 per plot). The germplasm of the 75 selected lines were stored and potentially become parental lines for the next cycles.
(7) F6: Line selection was conducted based on the total YA performance of all F6 individuals in each F4-line. Nine replicates were simulated for each F4-line, which means that each F6 line was grown in 9 plots. In total, 30 out of 75 lines with the highest YA were selected and were self-pollinated to produce 900 F7 per line (100 F7 per plot). The germplasm of the 30 selected lines were stored and they could potentially become parental lines for the next cycles.
(8) F7: Line selection was conducted based on the total YA performance of all F7 individuals in each F4-line. Nine replicates were simulated for each F4-line, which means that each F7 line was grown in 9 plots. In total, 5 out of 30 lines with the highest YA were selected and were self-pollinated to produce 900 F8 per line (100 F8 per plot). The germplasm of the five selected lines were stored and could potentially become parental lines for the next cycles.

**FIGURE 5:**
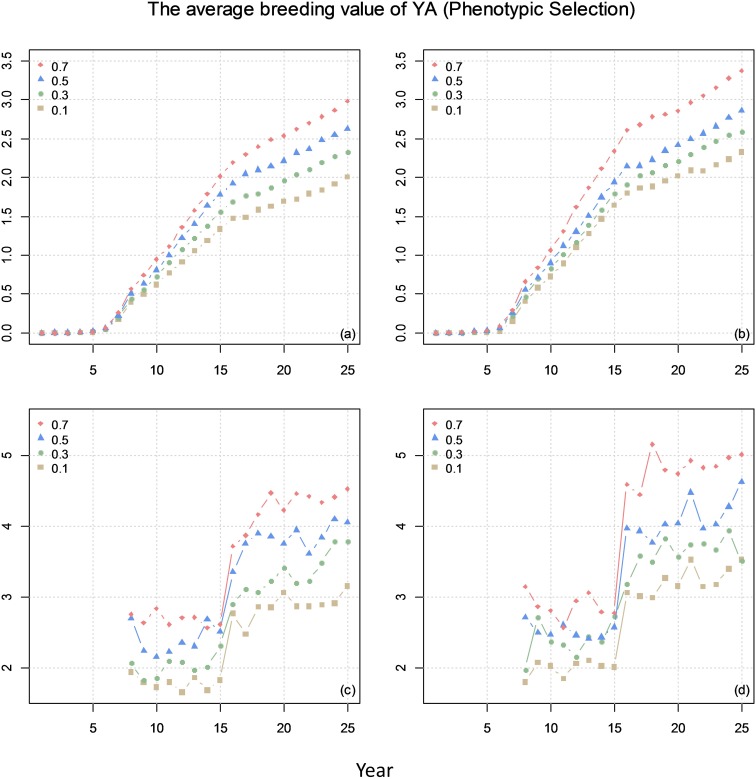
The average breeding value of yield at advanced yield trials (YA) of across the cycles every year (plots **a** and **b**) and the average breeding value of YA at F8 every year (plots **c** and **d**) from phenotypic selection with different genetic correlations between YA and yield at preliminary yield trial (YP). The correlation between BVP and the other two traits is 0 for **(a)** and **(c)** and 0.1 for **(b)** and **(d)**.

Figure 5 shows the average breeding value of YA across the cycles every year and the average breeding value of YA at F8 every year for different scenarios. The results show that higher correlation (positive) between the traits result in higher genetic gain.

### Breeding Plan B. Breeding Program With Genomic Selection

**FIGURE 6:**
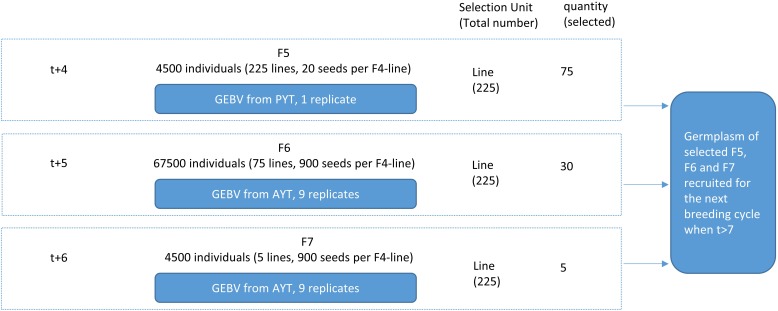
Overview of one cycle in the proposed breeding program with genomic selection (GS). Only the difference in the procedure of proposed breeding program with phenotypic selection is presented. PYT, preliminary yield trial; AYT, advanced yield trials.

**FIGURE 7:**
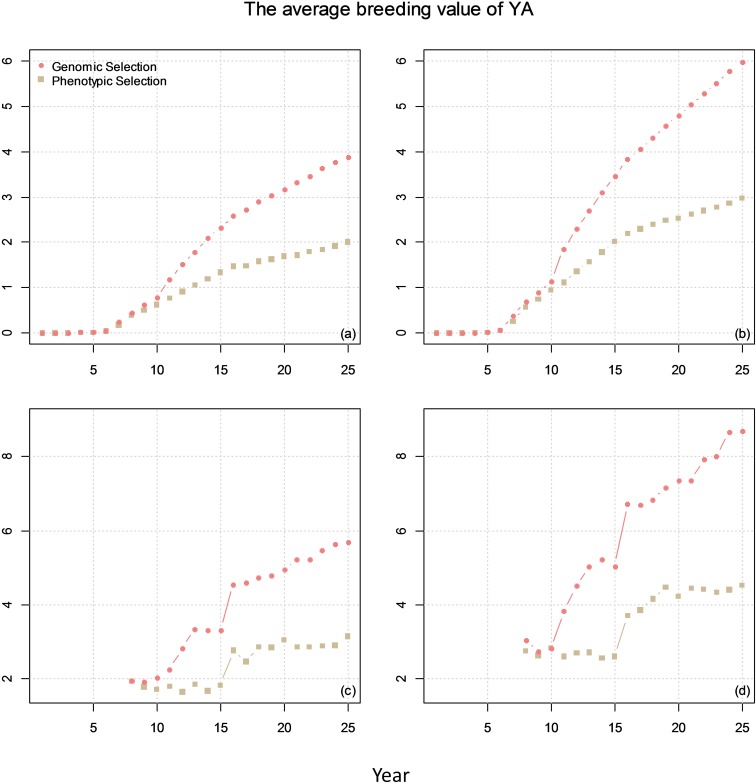
The average breeding value of YAs across the cycles every year (plots **a** and **b**) and the average breeding value of YA at F8 every year (plots **c** and **d**) from phenotypic selection and GS. In the figures, the correlation between BVP and the other two traits is 0. The genetic correlation between YA and YP is 0.1 for **(a)** and **(c)** and 0.7 for **(b)** and **(d)**.

For breeding plan B, a 25-year commercial wheat-breeding program using GS was simulated. The first 10 years was used as a burn-in stage, where PS was used as in breeding plan A without GS. The breeding strategy for this burn-in stage was the same as in breeding plan A. In the last 15 years, the selection decisions in F1 to F3 was the same as in breeding plan A. The current study present the breeding strategy of breeding plan B for F4–F7 at year 11–25 where the difference existed between breeding plan A and B (Figure 6):

(1) F4: The breeding strategy of F4 for GS was the same as for PS with the exception that all the 225 individual F4 were assumed genotyped in each cycle. The genotyped germplasm of F4 with phenotypes for the targeted traits were added yearly to establish the reference population (growing by 225 genotypes per year) for the current cycle where the selection is performed (Figure 7).

(2) F5: Line selection was conducted based on the YP performance of all F5 individuals in each F4-line. Only one replicate was simulated for each F4-line. YP performance of all F5 individuals within each F4-line was recorded, so that there were 225 phenotypes. These 225 phenotypes and 225 F4 genotypes and existing reference population were used to estimate breeding values of each F4 using GBLUP model (Madsen and Jensen, 2013), so that 75 highest-ranking F4-lines were identified given GEBVs of F4. In total, 75 out of 225 F5 lines with highest GEBV were selected and were self-pollinated to produce 900 F6 per F4 family (100 F6 per plot). The information of phenotypes and genotypes in the current cycle were stored and was used for predicting breeding values in the next cycles. The germplasm of the 75 selected lines were stored and potentially become parental lines for the next cycles.
(3) F6: Line selection was conducted for F6 based on the YP performance of all F5 individuals in each F4-line. Nine replicates was simulated, and therefore, each F4-line was grown in 9 plots. Similar to F5, total YA performance of all F6 individuals in each F4-line was recorded, so that there were 75 phenotypes. These 75 phenotypes and their corresponding F4 genotype were used for EBV estimation. In total, 30 out of 75 F6 lines with high GEBV were selected and were self-pollinated to produce 900 F7 per F4-line (100 F7 per plot). The germplasm of the 30 selected lines were stored and potentially become parental lines for the next cycles.
(4) F7: Line selection was conducted based on the total YA performance of all F7 individuals in each F4-line. Nine replicates were simulated for each F4-line, which means that each F7 line was grown in 9 plots. Similar to F5 and F6, five out of 30 lines with the highest GEBV were selected and were self-pollinated to produce 900 F8 per line (100 F8 per plot). The germplasm of the five selected lines were stored and potentially become parental lines for the next cycles.

The comparison of genetic gain from breeding plan A with that obtained from breeding plan B is given in Figure 7. The results showed that GS can double the genetic gain compared to PS.

### Breeding Plan C. Speed Breeding Program With Genomic Selection

For breeding plan C, a 25-year speed-breeding program for wheat using GS was simulated. The breeding program was similar to breeding plan B with the exception that the first 4 generations (F1-F4) were achieved within 1 year instead of 4 years.

**FIGURE 8:**
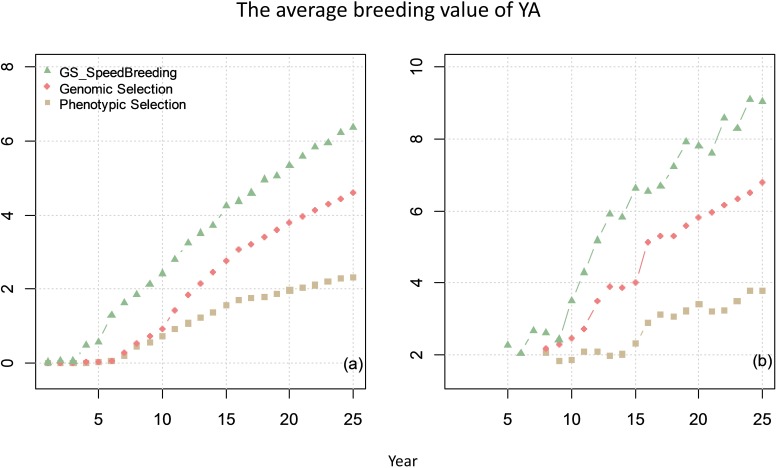
The average breeding value of YAs across the cycles every year (plot **a**) and the average breeding value of YA at F8 every year (plot **b**) from phenotypic selection, GS and speed breeding programs. In this scenario, the genetic correlation between yield at primary yield trial (YP) and YA is 0.3, and the genetic correlation between BVP and the other two traits is 0.

The comparison of genetic gain from breeding plan C with that obtained from breeding plan A and B is given in Figure 8. The results showed that speed-breeding program could markedly accelerate and increase the genetic gain.

The rate of genetic gain after the burn-in stage was the highest in breeding plan C with SPB (0.28–0.46), which was 2–2.5 times higher than breeding plan A with PS and was 7–20% higher than Brreding plan B with GS (Table 1). The total genetic variance was the highest with PS and the lowest in GS. The genetic variance was not influenced by the genetic correlation.

**Table 1:** The rate of genetic gain in generation 11–25 and genetic variance at generation 25 in yield at advanced yield trials (YA).

		**ΔG**			**Variance**	
		
Correlation^1^	PS	GS	SPB	PS	GS	SPB
0.1	0.08	0.26	0.28	1.48	0.54	0.57
0.3	0.13	0.28	0.37	1.48	0.55	0.63
0.5	0.14	0.32	0.42	1.48	0.53	0.65
0.7	0.15	0.38	0.46	1.45	0.54	0.62


*^*1*^The genetic correlation between yield at primary yield trial (YP) and YA. In all these scenarios, the genetic correlation between breeder’s visual preference (BVP) and the other two traits (YP and YA) is 0.*

All the three breeding plans were replicated 60 times and performed on a Linux server. The running time for each replicate was 2 h 15 ∼2 h 30 min for breeding plan A and B, and 6 h 40 min for breeding plan C.

## Discussion

ADAM-plant is a computer software for stochastic simulation of plant breeding programs that utilize diverse genomic and phenotypic information. It was developed with a purpose of guiding breeding decisions in early planning and implementation phases. To maximize its utility, it was developed based on effective models of the genome and the breeding process with great flexibility. It can simulate easy to very complicated breeding programs that are similar to commercial breeding plans (with GS and/or speed breeding technologies), and the best breeding strategy can be identified. Based on the results from simulation experiments, breeders have the opportunity to optimize their breeding methodology, and use of resources (number of matings, number of test plots, amount of genotyping etc.) and greatly improve breeding efficiency.

(1) There are a number of computer software packages available for simulating plant breeding programs. However, ADAM-plant has several advantages over the others: It allows users to simulate overlapping breeding cycles with the possibility of a new cycle starting at each time step. The simulation of overlapping breeding cycles allows information and elite genetic material transfer from one breeding cycle to another. It means that early generations in one cycle can be used as parents in a new cycle. This system resembles the procedure of actual commercial breeding programs for crop plants. Adam-Plant also enables simulation of breeding programs in competing companies, which use a proportion of parental lines from the other companies.
(2) It allows simulation of speed breeding with flexibilities in defining generation intervals in order to test the effectiveness of selection in early generations and to quantify genetic progress and genetic variance using different designs for speed breeding. It is also possible to test the potential for integrating speed breeding with GS in accelerating the rate of genetic improvement in simulated crop breeding programs.
(3) It allows storing the advanced germplasm in any generation and these germplasm can be used for later cycles. This function makes it more flexible for selection of parental lines.
(4) There are more options for phenotyping, genotyping strategies, selection and mating strategies. For instance, optimum contribution selection or minimum co-ancestry mating can be carried out for cross-pollinated crops such as maize in order to constrain inbreeding while ensuring high genetic progress by managing the distribution of genetic contributions to the selected candidates.
(5) It allows different units for selection. The selection unit can be population, within family, or entire family, in which the family can be defined with great flexibility e.g., a set of parents or a set of plants used in a poly-cross to create synthetics) in an earlier generation.
(6) Adam-plant allows great flexibility in mating strategies as it allows crossing of individuals that are in different generations or in different selection units. For instance, ADAM-plant allows backcrossing by storing the germplasm of one of the parent and crossing this parent to its offspring in the generation where selection is performed, it allows three way cross by crossing an inbred line to an F1, it allows crossing of two single crosses that come from four separate inbred parents and it allows crossing between the individual plants within a pre-defined unit i.e., full-sib cross or half-sib cross.

For the three examples presented, breeding plan A represented a traditional breeding program in wheat. Breeding plan B represented a modern breeding program, which is a combination of conventional breeding techniques and genomic tools leading to a new genomics-based plant breeding. Breeding plan C represented a new technology for rapid generation advance named “speed breeding,” which has been successfully deployed in bread wheat (Alahmad et al., 2018).

The comparison between breeding plans A and B indicated that even adding 2000 genotypes per breeding cycle could result in 2 times higher genetic gain. Phenotypic selection resulted in higher genetic variance mainly due to less fixation of favorable alleles with less accurate selection. Compared with breeding plan 2, breeding plan 3 maintained more genetic variance as well as resulted in higher genetic gain because the parent lines were updated more rapidly. In the current study, real haplotypes were used as parental lines considering the real LD patterns in a commercial wheat-breeding program, and the schemes simulated were similar to current commercial breeding programs. Therefore, these results indicate that combining speed breeding with GS is a very promising tool in plant breeding. When the speed breeding and GS are considered, the initial facilities investment such as a growth chamber with appropriate supplemental lighting and temperature control capabilities can be substantial (Ghosh et al., 2018). However, light-emitting diode supplemental lighting with more efficient power usage and decreasing cost of sequencing for a small number of single individuals provides significant cost saving, and these costs may also be compensated by a rapid genetic improvement and the benefit can be expected after a short time if the facilities are constructed (Ghosh et al., 2018; Watson et al., 2018).

ADAM-plant is fast in generating simple breeding programs and genotype data. However, the time consumed can become significantly larger in particular when a large number of plants and complex breeding strategies are simulated. The computation time also depends on the utilization of genomic information and on the genetic model used. With polygenic model where no sequence data is generated, the running time is short even with complex selection steps and large number of seeds. When a very precise prediction is required with consideration of full genomic information, however, the time is markedly increased due to sampling of molecular information (QTLs, markers and crossovers in the genome) and so on for each single plant. For example, one replicate of a 40 year – commercial GS breeding program with 40 overlapping breeding cycles takes around 23 h for running (Tessema et al., unpublished data, 2018).

The software was developed with modules, which makes it easy to be extended with new methodologies for example on how to utilize genomic information. Although the examples presented were wheat breeding programs, ADAM-plant can also be used for simulating many other plant species such as outcrossing crop maize and diploid ryegrass. In the future, the breeding program design for tetraploid plants will be integrated in the software. Dominance effects that are important for cross-pollinated crops and epistasis effects that are important for self-pollinated crops will also be integrated.

## Conclusion

In conclusion, ADAM-plant is a flexible and efficient computer software for stochastic simulation of breeding plans for crop plants. ADAM-plant simulate real commercial breeding program structures with parallel breeding cycles, GS and speed breedingfor self-pollinated and cross-pollinated plant crops. This makes ADAM-plant an important tool to compare breeding efficiencies and the improvement of performance from a wide range of selection strategies.

## Author Contributions

HL and AS developed the software. HL performed the analyses and prepared the manuscript. BT, AS, JJ, FC, and JA helped to design the programs, participated in interpretation of results and revision of the manuscript. All authors read and approved the final manuscript.

## Conflict of Interest Statement

The authors declare that the research was conducted in the absence of any commercial or financial relationships that could be construed as a potential conflict of interest.

## Footnotes

1 http://sites.google.com/view/qu-gene

## Abbreviations

BLUP: best linear unbiased prediction
BVP: breeder’s visual preference
DHs: doubled-haploids
EBV: Estimated breeding value
GEBV: genomic estimated breeding value
GS: genomic selection
IBD: identical-by-descent
LD: linkage disequilibrium
MAS: marker-assisted selection
PS: phenotype-based selection
QTL: quantitative trait locus
TBV: true breeding value
YA: yield at advanced yield trial
YP: yield at preliminary yield trial
